# The utility of magnetic resonance imaging in prostate cancer diagnosis in the Australian setting

**DOI:** 10.1002/bco2.99

**Published:** 2021-06-04

**Authors:** Jia Ying Isaac Tay, Ken Chow, Dominic J. Gavin, Evie Mertens, Nicholas Howard, Benjamin Thomas, Philip Dundee, Justin Peters, Paul Simkin, Sevastjan Kranz, Moira Finlay, Stefan Heinze, Brian Kelly, Anthony Costello, Niall Corcoran

**Affiliations:** ^1^ Department of Urology The Royal Melbourne Hospital Melbourne VIC Australia; ^2^ Department of Surgery The Royal Melbourne Hospital Melbourne VIC Australia; ^3^ Department of Radiology The Royal Melbourne Hospital Melbourne VIC Australia; ^4^ Department of Pathology The Royal Melbourne Hospital Melbourne VIC Australia

**Keywords:** active surveillance, biopsy, ERSPC risk calculator, multiparametric magenetic resonance imaging, prostate Cancer

## Abstract

**Objectives:**

To investigate the utility of Magnetic Resonance Imaging (MRI) for prostate cancer diagnosis in the Australian setting.

**Patients and methods:**

All consecutive men who underwent a prostate biopsy (transperineal or transrectal) at Royal Melbourne Hospital between July 2017 to June 2019 were included, totalling 332 patients. Data were retrospectively collected from patient records. For each individual patient, the risk of prostate cancer diagnosis at biopsy based on clinical findings was determined using the European Randomized study of Screening for Prostate Cancer (ERSPC) risk calculator, with and without incorporation of MRI findings.

**Results:**

MRI has good diagnostic accuracy for clinically significant prostate cancer. A PI‐RADS 2 or lower finding has a negative predictive value of 96% for clinically significant cancer, and a PI‐RADS 3, 4 or 5 MRI scan has a sensitivity of 93%. However, MRI has a false negative rate of 6.5% overall for clinically significant prostate cancers. Pre‐ biopsy MRI may reduce the number of unnecessary biopsies, as up to 50.0% of negative or ISUP1 biopsies have MRI PI‐RADS 2 or lower. Incorporation of MRI findings into the ERSPC calculator improved predictive performance for all prostate cancer diagnoses (AUC 0.77 vs 0.71, *P* = .04), but not for clinically significant cancer (AUC 0.89 vs 0.87, *P* = .37).

**Conclusion:**

MRI has good sensitivity and negative predictive value for clinically significant prostate cancers. It is useful as a pre‐biopsy tool and can be used to significantly reduce the number of unnecessary prostate biopsies. However, MRI does not significantly improve risk predictions for clinically significant cancers when incorporated into the ERSPC risk calculator.

## 1 INTRODUCTION

Prostate cancer is a prevalent disease, and case finding for clinically significant prostate cancer is an important population health focus. In Australia, this involves the use of a clinical evaluation including family history and digital rectal examination, followed by Prostate Specific Antigen (PSA) testing in the community setting.^1^ However, PSA lacks specificity as it is commonly elevated in many benign conditions and may lead to unnecessary, invasive biopsies. This leads to cancer overdiagnosis particularly clinically indolent ones. There is an estimated overdiagnosis of 41% in Australia, 43% in Europe and 42% in the United States.^2^, ^3^, ^4^ Well differentiated cancers with low metastatic potential carry minimal risk of symptomatic progression or mortality, but their detection may lead to overtreatment with radical surgery or radiation, which in turns comes with risks such as urinary incontinence and erectile dysfunction.^5^ The challenge hence lies in improving our current evaluation strategies to be able to distinguish clinically significant from indolent cancer.

Magnetic Resonance Imaging (MRI) was first used in 1982 to study prostate cancer. As its accuracy improved with technical enhancements and the addition of functional parameters, multiparametric MRI has been increasingly used in the assessment of prostate cancer risk. Prostate Imaging Reporting and Data System (PI‐RADS) V2 was established to grade MRI findings from 1 to 5, correlating to the likelihood of clinically significant cancer.^6^ Several studies have validated the use of MRI in prostate cancer diagnosis, with good sensitivity and negative predictive value for clinically significant disease.^7^, ^8^, ^9^

The lack of specificity of PSA as well as the risk of overdiagnosis of indolent cancers, has led to the development of personalized clinical prostate cancer risk calculators, which increasingly incorporate the findings of pre‐biopsy prostate MRI. The European Risk Surveillance for Prostate Cancer (ERSPC) is one such risk calculator that generates two risk predictions, one for all prostate cancers and one for clinically significant cancers, based on information such as age, DRE, PSA, prostate volume, and previous negative biopsies. Clinically significant cancer is defined as having a Gleason score equal or greater than 7.^10^ Based on the RCT by Roobol et al, the risk calculator recommends that a risk prediction less than 12.5% does not require a biopsy, and a risk of more than 20% warrants a definite biopsy.^11^ For percentages in between, the decision to biopsy depends on co‐morbidities and if the risk for clinically significant cancer was more than 4%. In an international study involving ten cohorts in Europe and Australia, ERSPC was shown to have the highest predictive accuracy, in comparison to other risk calculators such as Sunnybrook and Prostate Cancer Prevention Trial (PCPT) risk calculator.^12^ Furthermore, there have been improvements with a newer edition that allows the incorporation of MRI results in recent years.

Australia has the highest incidence of prostate cancer diagnoses globally, and as such it is plausible that findings from international studies may be less applicable in our local context. Limited data from a small number of studies suggest that the performance of the test is similar to that observed internationally, with the presence of an MRI abnormality being significantly associated with the presence of clinically significant disease.^13^, ^14^, ^15^ However, these studies were performed prior to MRI being universally funded, and so estimates of performance may be biased by patient selection. Since the new Medicare Benefit Schedule was rolled out in Australia 2018, prostate MRI has become rebatable if the patient met eligibility criteria, such as having positive DRE, two serial elevated PSA and a free‐to‐total PSA ratio of less than 25%, or undergoing active surveillance.^16^ With the increasing use of MRI in the Australian community, it is vital to study and understand the utility of MRI. This will impact how we can appropriately incorporate these findings into the risk stratification of our prostate cancer patients. In this study we evaluated the performance of prostate MRI in the pre‐diagnostic setting in patients meeting funding current criteria, and asses its incremental diagnostic utility when incorporated into the established ERSPC clinical risk calculator.

## 2 PATIENTS AND METHODS

### 2.1 Patient selection

All consecutive men who underwent a prostate biopsy (transperineal or transrectal ultrasound‐guided) at Royal Melbourne Hospital between June 2017 to June 2019 were included in our study. These patients were identified from our internal Theatre List systems based on MBS coding. Patient demographic as well as clinicopathological data were retrospectively collected from the prospectively recorded health information record. Data collected included age at biopsy, family history of prostate cancer, PSA, digital rectal examination findings, previous biopsy results, prostate volume, and biopsy surgery type. Histopathology results were collected from the AUSCARE pathology system. Pathology results collected included the histopathological result of the biopsy and prostatectomy, and its Gleason score & International Society of Urologic Pathologists (ISUP) Grade, number of cores involved, and the involvement of margins for prostatectomy. Radiology information was derived from scan results on Synapse Picture Archiving and Communicating System (PACS). These data were de‐identified and entered into a secure electronic database. This study was approved by the Melbourne Health Research Ethics Committee (approval no. QA2020011).

### 2.2 Imaging

Prostate multiparametric MRI scans were performed using 3‐Tesla magnets Siemens Prisma and Skyra MRIs. T1‐ and T2‐weighted imaging, diffusion‐weighted imaging and apparent‐diffusion coefficient functional sequences were acquired and processed with Fujifilm Synapse PACS. Images were reported using the PI‐RADS v2 criteria by radiologists with expertise in MRI and body imaging. In our study, data were collected from 2017, a year after PI‐RADS v2 was adopted locally in Royal Melbourne Hospital to minimize the initial learning curve.

### 2.3 ERSPC risk calculator

The 3rd and 4th versions of the ERSPC calculator allow for the incorporation of MRI results.^10^ Using this calculator in our study allows us to compute and compare prostate cancer risk values derived with and without MRI incorporation, hence evaluating prostate cancer diagnosis accuracy of MRI. Each patient and his clinical, radiological and histopathology information is entered into the ERSPC risk calculator. Based on the patient profile, the risk calculator generates two risk percentages of all prostate cancers and clinically significant prostate cancer. The percentage values allow direct comparison and further statistical analysis of whether MRI‐incorporated ERSPC or non‐MRI incorporated ERPSC prediction model was more accurate for predicting prostate cancer. The risk data were collected by one author (JT), and a random sample of 20% of the data was cross‐checked independently by another author (DG).

### 2.4 Statistical analysis

All data were analyzed and presented with simple descriptive statistics. Categorical data were compared using chi‐square test and continuous variables using the student *t*‐test. The MedCalc statistical software was used to calculate and generate the Receiver Operator Curve (ROC). Thereafter, Area Under Curve was calculated using the Hanley and McNeil algorithm. All statistical tests were 2‐sided with *P* < .05 considered statistically significant.

## 3 RESULTS

In total, 332 patients were identified, the clinical, radiological and pathological characteristics of whom are summarized in Table 1. The average patient in our study was 64.6 years old at the date of biopsy, with a PSA of 6.6 ng/mL and a prostate volume of 40 cc. The majority of patients had a normal DRE, no family history of prostate cancer, and no previous biopsy. Sixty‐two percent of the patients had an MRI scan of their prostate, of which 40.3% of the scans were PI‐RADS 2 or lower and 59.7% were PI‐RADS 3 and above. The majority of patients underwent TP biopsy. There were almost equal proportions of benign and malignant biopsy results; 51.0% negative and 49.0% cancers. Of the cancers diagnosed, 41.1% were ISUP grade group 1, 25.8% were ISUP grade group 2 and 33.1% were ISUP grade group 3 and above. Metastatic disease was detected in 6.7% of the cohort. In terms of management, 41.1% had active surveillance or watchful waiting and 7.4% had palliative hormonal and/or chemotherapy. The remainder were actively treated, of which 62.7% underwent radical prostatectomy and 37.3% received radiotherapy and/or hormonal therapy. Of the patients who underwent radical prostatectomy, 3.9%, 53.9% and 42.2% were found to have ISUP1, ISUP2 and ISUP 3 or above, respectively, on final pathological review. Patients without MRI were significantly older, with higher PSAs and higher rates of high grade and metastatic disease, likely reflecting the patient factors that contribute to investigating with an MRI, rather than any actual clinically significant difference between the 2 groups.

**TABLE 1:** Clinical, radiological and pathological characteristics of study cohort

Variable	Total	Patients without MRI	Patients with MRI	*P* value
MRI, n (%)	332 (100.0)	126 (38.0)	206 (62.0)	–
Age at biopsy, years, median (IQR)	65 (58.5‐69)	65 (59‐71)	64.0 (58‐68)	.026
PSA level, ng/mL, median (IQR)	6.6 (4.7‐9.5)	7.2 (4.8‐12.5)	6.35 (4.6‐9.0)	.017
Family history, n (%)				
Yes	47 (14.2)	12 (9.5)	35 (17.0)	.058
No	285 (85.8)	114 (90.5)	171 (83.0)	
Digital rectal examination, n (%)				
Normal	223 (67.2)	78 (61.9)	145 (70.4)	.094
Abnormal	104 (31.3)	47 (37.3)	57 (27.7)	
Not recorded	5 (1.5)	1 (0.8)	4 (1.9)	
Previous biopsy, n (%)				
Yes	98 (29.5)	30 (23.8)	68 (33.0)	.075
No	234 (70.5)	96 (76.2)	138 (67.0)	
MRI PI‐RADS, n (%)				
PI‐RADS ≤2	83 (40.3)	–	83 (40.3)	–
PI‐RADS 3	41 (19.9)		41 (19.9)	
PI‐RADS 4	51 (24.8)		51 (24.8)	
PI‐RADS 5	31 (15.0)		31 (15.0)	
Volume of prostate, cc, median (IQR)	40.0 (31.7‐59.0)	38.0 (30.0‐50.0)	45 (33.0‐61.0)	.070
Derivation of prostate volume, (%)				
TRUS	280 (84.3)	113 (89.7)	167 (81.0)	<.001
MRI	36 (10.9)	0	36 (17.5)	
DRE	1 (0.3)	1 (0.8)	0	
Not recorded	15 (4.5)	12 (9.5)	3 (1.5)	
Prostate biopsy type, n (%)				
Transperineal biopsy	213 (64.2)	54 (42.9)	159 (77.2)	<.001
Transrectal ultrasound‐guided biopsy	119 (35.8)	72 (57.1)	47 (22.8)	
Biopsy results, n (%)				
Benign	169 (51.0)	61 (48.4)	108 (52.4)	.478
Cancer	163 (49.0)	65 (51.6)	98 (47.6)	
Biopsy ISUP grade group, n (%)				
1	67 (41.1)	15 (23.1)	52 (53.1)	<.001
2	42 (25.8)	17 (26.1)	25 (25.5)	
3	22 (13.5)	12 (18.5)	10 (10.2)	
4	14 (8.6)	7 (10.8)	7 (7.1)	
5	18 (11.0)	14 (21.5)	4 (4.1)	
Metastatic disease, n (%)				
Yes	11 (6.7)	10 (15.4)	1 (1.0)	<.001
No	152 (93.3)	55 (84.6)	97 (99.0)	
Treatment, n (%)				
Active surveillance/watchful waiting	67 (41.1)	19 (29.2)	48 (49.0)	.001
Radical prostatectomy	52 (31.9)	21 (32.3)	31 (31.6)	
Curative hormonal/radiotherapy	31 (19.0)	22 (33.9)	9 (9.2)	
Palliative hormonal/chemotherapy	12 (7.4)	3 (4.6)	9 (9.2)	
Lost to follow up	1 (0.6)	0	1 (1.0)	
Prostatectomy ISUP grade group, n (%)				
1	2 (3.9)	1 (4.8)	1 (3.2)	<.001
2	28 (53.9)	9 (42.9)	19 (61.3)	
3	10 (19.2)	4 (19.0)	6 (19.4)	
4	6 (11.5)	4 (19.0)	2 (6.4)	
5	6 (11.5)	3 (14.3)	3 (9.7)	

MRI PI‐RADS grading corresponded well with the biopsy results, especially with clinically significant cancers. The proportions of patients with each PI‐RADS and ISUP grade are summarized in Table 2. All ISUP 3 to 5 biopsies were predominantly PI‐RADS 4 and 5, with only 14.3% of ISUP 4 cancers having PI‐RADS 3 findings on MRI. The majority of ISUP 2 cancers (88.0%) were PI‐RADS 3 or above on imaging. For PI‐RADS 2 and below MRI results, there were 73.5% negative biopsies, 22.9% ISUP 1, and 3.6% ISUP 2 biopsies. In terms of diagnostic accuracy, a PI‐RADS 2 or lower MRI finding has a high negative predictive value of 0.96 for clinically significant cancer (ISUP2 or higher). On the other hand, a PI‐RADS 3, 4 or 5 MRI scan has a sensitivity of 0.93 for cancers ISUP 2 and above. The positive predictive value and specificity were 0.35 and 0.50, respectively.

**TABLE 2:** Absolute number and proportion of patients characterised into PI‐RADS and ISUP grades

ISUP	PI‐RADS			
	PI‐RADS ≤2	PI‐RADS 3	PI‐RADS 4	PI‐RADS 5
Neg	61 (29.6%)	28 (13.6%)	16 (7.8%)	3 (1.5%)
1	19 (9.2%)	10 (4.8%)	17 (8.2%)	6 (2.9%)
2	3 (1.5%)	2 (1.0%)	9 (4.4%)	11 (5.3%)
3	0	0	4 (1.9%)	6 (2.9%)
4	0	1 (0.5%)	4 (1.9%)	2 (1.0%)
5	0	0	1 (0.5%)	3 (1.5%)

Looking at our dataset, 77.7% of all prostate biopsies were ISUP 1 or negative, of which 50.0% of them had MRI findings of PI‐RADS 2 or lower. Of all the clinically significant cancers, 93.5% had PI‐RADS 3 and above lesions, and 6.5% of them had PI‐RADS 2 or lower. These 6.5% of clinically significant cancers were all ISUP 2 cancers, with less than 5% pattern 4 disease and an average maximum cancer core length of 6 mm.

**FIGURE 1:**
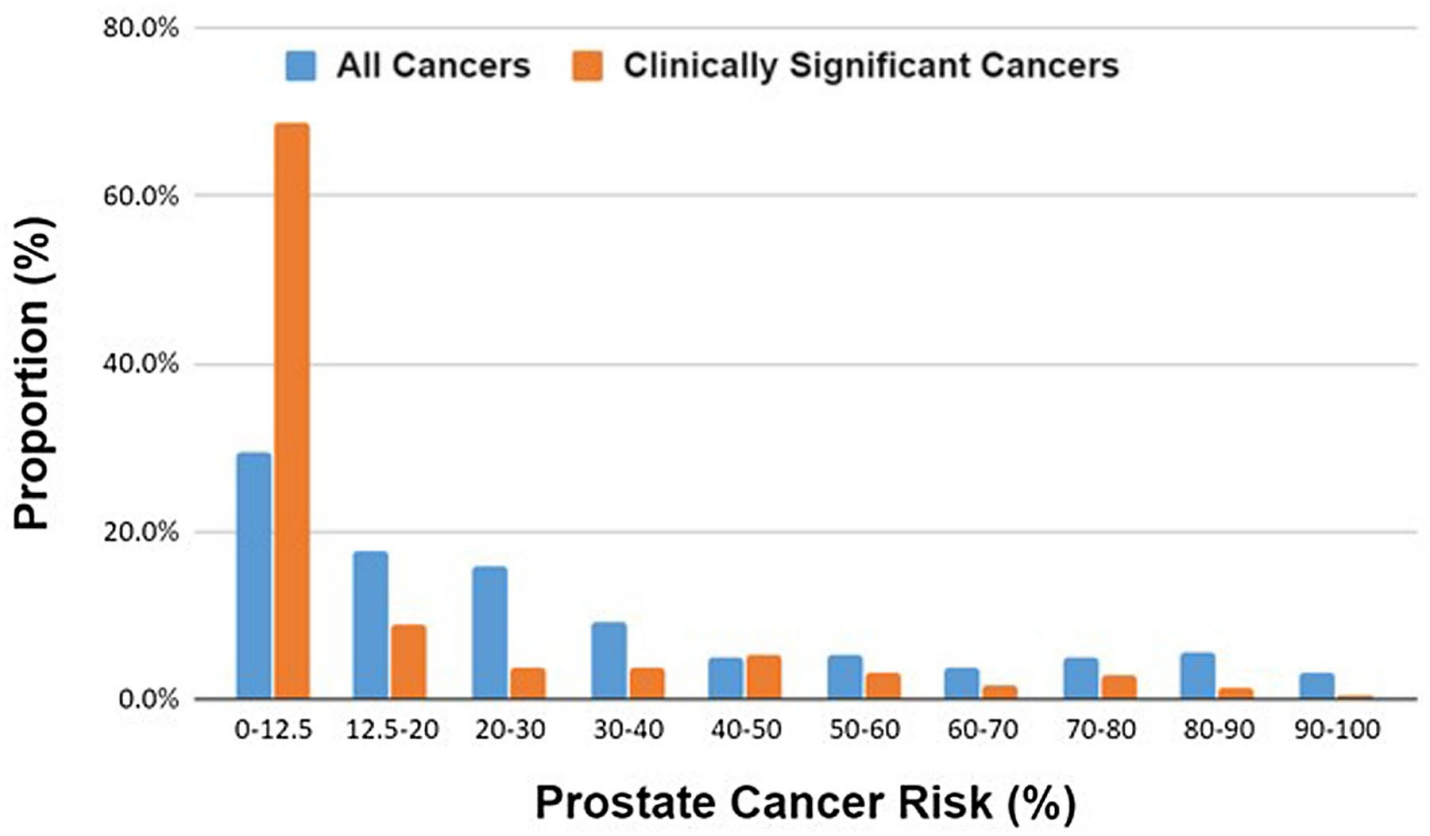
Distribution of prediction risk scores for clinically significant and all prostate cancers

Figure 1 depicts the distribution of ERSPC prediction risk scores in our cohort, where significant skewing to lower risk scores was observed. We found that 29.5% of our patients had a risk of less than 12.5% for all prostate cancers, where a prostate biopsy is not recommended by the studies that validated the risk calculator.

**FIGURE 2:**
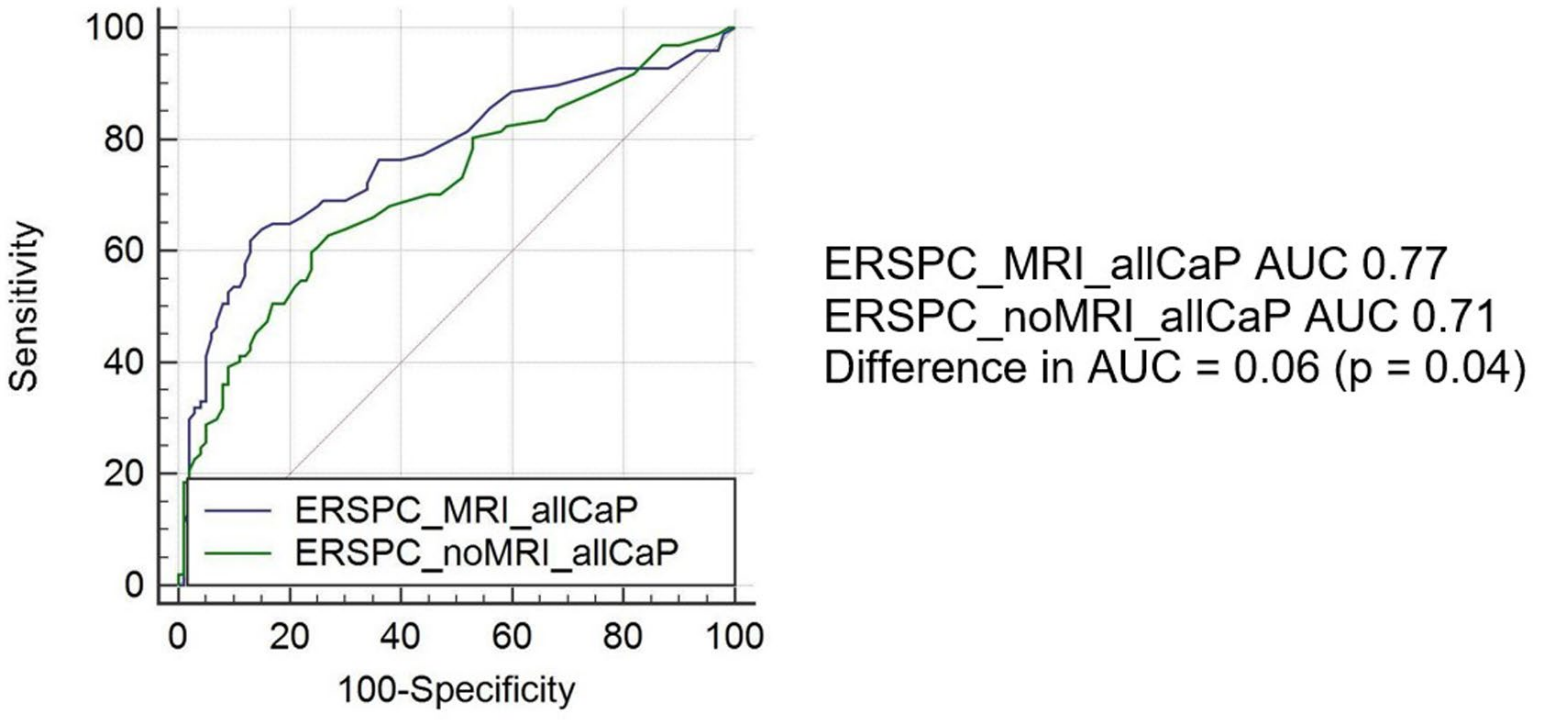
Receiver operator curve of ERSPC prediction of all prostate cancer risks with MRI and without MRI

**FIGURE 3:**
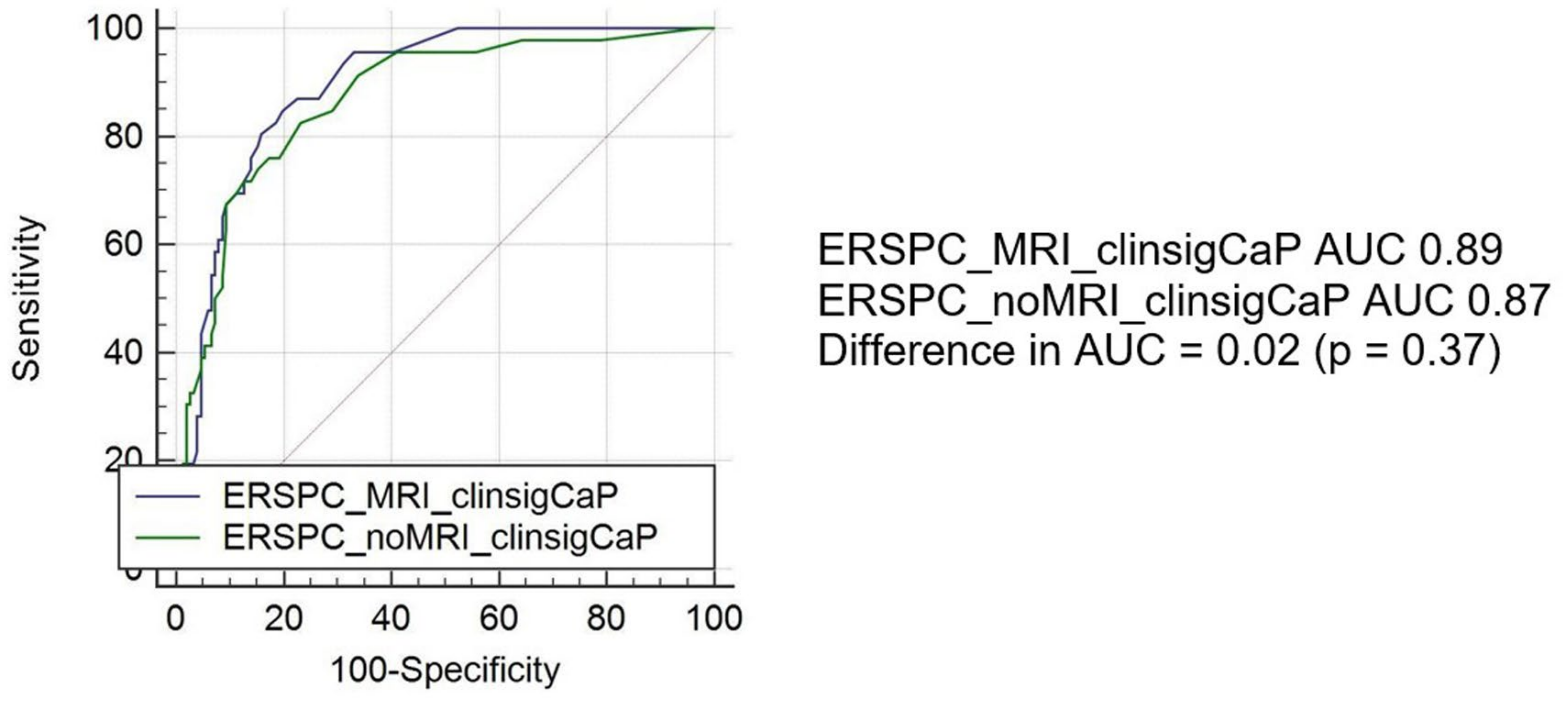
Receiver operator curve of ERSPC prediction of clinically significant prostate cancer risks with MRI and without MRI

Figures 2 and 3 represent the Receiver Operator Curve of the ERSPC predictions for all and clinically significant prostate cancers, respectively, and their calculated AUC. The AUC for MRI‐incorporated ERSPC prediction for all prostate cancer risks is 0.77 (95% CI 0.71‐83). This is in comparison with an AUC of 0.71 (95% CI 0.64‐0.77) when MRI is not used (*P* = .04). The AUC for the MRI‐incorporated ERSPC prediction for clinically significant prostate cancer risks for biopsy results is 0.89 (95% CI 0.84‐0.93). This is in comparison with 0.87 (95% CI 0.82‐0.91) when MRI is not used (*P* = .37).

Overall, 51.9% of prostate biopsy results had the same ISUP grades as the final prostatectomy histopathology, as seen in Table 3. Of the remaining discordant results, 72.0% had upgrading. From our study, the use of pre‐biopsy MRI does not appear to increase the concordance of the biopsy results and final histopathology (*P* = .19). However, the sample is small and larger numbers will be needed to reliably interpret this finding.

**TABLE 3:** Concordance rates of biopsy and prostatectomy ISUP results, with and without pre‐biopsy MRI

	With pre‐biopsy MRI	Without pre‐biopsy MRI	Total
Concordant results	13 (43.3%)	14 (63.6%)	27 (51.9%)
Discordant results	17 (56.7%)	8 (36.4%)	25 (48.1%)

## 4 DISCUSSION

There is limited data on the use of MRI in the screening and diagnosis of prostate cancer in Australia. Our results have shown that MRI has good diagnostic accuracy for clinically significant prostate cancer. A PI‐RADS 2 or lower finding has a high negative predictive value of 96% for clinically significant cancer and a PI‐RADS 3 or above lesion has a sensitivity of 93%. This is similar to the findings from other international studies which ranges from 79%‐100% and 76%‐100%, respectively.^7^, ^8^, ^9^

MRI may reduce the number of unnecessary biopsies. In our retrospective study, 50.0% of ISUP 1 or negative biopsies had PI‐RADS 2 or below findings, which reflects 38.8% of the total biopsies. This is similarly reflected with the ERSPC risk predictions, where 29.5% of our cohort would be recommended not to proceed to biopsy. If MRI was used universally as a risk stratification tool, the reduction of around a third of biopsy procedures is of great significance in reducing healthcare spending in terms of the procedural cost, peri‐operative investigations and clinic reviews. It can also reduce the waiting lists to ensure better and more timely allocation of services and reduce side effects from biopsies and unwarranted patient anxiety over false positive results.

There remains concern that MRI can miss some clinically significant prostate cancers. In our study, 6.5% of clinically significant prostate cancers had PI‐RADS 2 findings or lower, which is similar to the PROMIS study, where there were 11% MRI‐negative clinically significant cancers found on TP biopsy.^9^ However, in our study, these cancers were all low‐volume ISUP 2 disease. A meta‐analysis of 21 studies by Kane et al demonstrated that the 5‐year progression free rate of ISUP 2 cancers to be 88%, which was similar to GS 6, as opposed to 63% for ISUP 3. The cancer specific survival was 92.1% and 76.5% for ISUP 2 and 3 cancers, respectively.^17^ Multiple other studies also found no difference in outcomes in this group of low‐volume ISUP 2 cancers, suggesting that they can managed with active surveillance.^18^, ^19^, ^20^, ^21^, ^22^ Therefore, the false negative results might be mitigated by close follow‐up, although this may offset some of the savings gained by reducing the number of biopsies.

Our study also showed that the ERSPC risk calculator is well validated for an Australian population. For non‐MRI ERSPC risk prediction, the AUC was 0.71 for all cancers and 0.87 for clinically significant cancers, which on average is similar to the 10‐centres study by Pereira‐Azevedo et al with an AUC of 0.77.^12^ The inclusion of MRI results in the newer versions of the ERSPC risk calculator, further improved its risk prediction. A 2019 study in Germany and Holland found that with the incorporation of MRI results, the AUC increased from 0.74 (95% CI 0.69‐0.79) to 0.85 (95% CI 0.81‐0.89) for previously biopsied men, and from 0.76 (95% CI 0.72‐0.80) to 0.84 (95% CI 0.81‐0.88) for biopsy‐naive men.^23^ This is similar in our study, where the AUC improved to 0.77 for all cancers and 0.89 for clinically significant cancers when MRI was incorporated. There have not been any other studies showing the external validity of ERSPC risk calculator for Australian men in predicting prostate cancer. Pereira‐Azevedo et al compared different risk calculators in their prediction accuracy in ten independent cohorts, of which nine are European and one is Australian.^12^ No specific analysis was done for the independent cohorts, and hence the data for validation is not specific for Australia.

As seen in Figure 1, our cohort is skewed towards having low calculated risk scores, with almost a third of patients having risk scores less than 12.5%. This could be attributed to the use of free‐to‐total PSA ratio in the Australian context, as this is one of the pre‐requisite tests to obtaining a Medicare‐funded MRI.^16^ A ratio of less than 25% was associated with an increased risk of prostate cancer, but the risk is still low at 16%‐20% for a free‐to‐total ratio from 15% to 25%.^24^ The regular monitoring of this ratio could have prompted biopsy in patients who might otherwise have low risk of prostate cancer. This could contribute to the findings of low‐risk scores in our cohort. The use of free‐to‐total ratio is not included in the ERSPC predictions.

Our study has some limitations. Firstly, the retrospective nature of this study precludes a standardized and more robust follow‐up. There was also incomplete incorporation of MRI data into the algorithm as not every patient had MRI scans. A prospective and randomized trial would also be able to provide more control and more direct analysis without biases. Our study was based on screening assessment of a patient population from a single institution and the findings may not be consistent with other worldwide patient populations or institutions. As a referral centre, many of the patients also who attended RMH for biopsies ended up having their definitive treatment in other centres, hospitals or private practices, which contributed to the paucity of follow up data. A larger sample size will also be more statistically powered to give stronger recommendations. The utility of multiparametric MRI could also be evaluated further with the use of current fusion technology with other imaging modalities. Future studies could also analyze the utility of MRI in the different subgroups such as a post biopsy surveillance tool or for a biopsy‐naive patient.

## 5 CONCLUSION

The use of MRI has been increasing in the last few years, especially in Australia following the implementation of healthcare rebate policies. Our study shows that MRI is useful as a pre‐biopsy tool, as it can significantly reduce the number of unnecessary prostate biopsies. It improves the risk prediction of clinically significant cancers and has good sensitivity and negative predictive value. However, the incremental improvement over the ERSPC risk calculator is low.

## CONFLICT OF INTEREST

The authors have nothing to disclose, and there is no conflict of interest to declare. This paper is not based on communication to a society or meeting.
